# Renal arteriovenous malformation causing hematuria: Case report and review of the literature

**DOI:** 10.1097/MD.0000000000034547

**Published:** 2023-08-25

**Authors:** Xin Wang, Zhankui Zhao

**Affiliations:** a Clinical Medical College, Jining Medical University, Jining, China; b Department of Urology, Affiliated Hospital of Jining Medical University, Jining, China.

**Keywords:** hematuria, renal arteriography, renal arteriovenous malformations, renal artery embolization, vascular morphological anomalies

## Abstract

**Rationale:**

Renal arteriovenous malformations are rare vascular morphological anomalies that can be classified as congenital, idiopathic and acquired, of which congenital renal arteriovenous malformations are the most common. This disease is a rare cause of hematuria. In this case report, we report the diagnosis and treatment of a patient with renal arteriovenous malformation. We also review the symptoms, diagnosis and treatment of renal arteriovenous malformations in the published literature.

**Patient concerns:**

A 35-year-old female patient presented to a local hospital with right-sided lumbar abdominal pain with hematuria for 2 days. Physical examination showed percussion pain in the right renal area. Laboratory tests such as routine blood and blood biochemistry did not show any significant abnormalities when the patient entered the hospital. Considering the patient’s medical history, a urological computed tomography scan showed blood accumulation in the right renal pelvis, upper middle ureter and bladder. Subsequently, routine blood tests showed that the patient’s red blood cells and hemoglobin continued to decrease. An emergency renal arteriogram was performed, which showed a tortuous right upper renal pole branch artery and multiple thickened veins communicating with it.

**Diagnosis:**

This patient was diagnosed with cirsoid renal arteriovenous malformation.

**Interventions:**

Renal artery embolization was performed immediately after the renal arteriogram was performed on the patient.

**Outcomes:**

On review of the angiogram, the tortuous right upper renal pole branch artery was found to be obstructed, and the thickened vein disappeared, and the renal vein was normally visualized in due course. On the third postoperative day, the patient was free of hematuria. Physical examination showed no percussion pain in the renal area. The patient healed and was discharged. A 1-year follow-up was performed and the patient gave feedback that she no longer had symptoms such as back pain and hematuria in her daily life.

**Lessons:**

This case illustrates that early use of vascular interventions is an important method for the diagnosis and treatment of cirsoid renal arteriovenous malformations.

## 1. Introduction

Renal arteriovenous malformations (rAVMs) are rare abnormal vascular pathways between the renal arteries and veins. The arteries and veins within the kidney are not connected by capillary structures, but by an abnormal network of tortuous and dilated vessels, through which arterial blood passes directly into the veins.^[1]^ rAVMs are classified as congenital, idiopathic, and acquired. The main clinical symptom of the disease is hematuria of the naked eye, which is most often seen in congenital rAVMs. Congenital rAVM can be divided into the cirsoid, angiomatous, and aneurysmal types based on the structure of the vessels. The cirsoid type is the most common and has multiple arteriovenous interconnected varicose vascular communications. The angiomatous type is characterized by a single large artery feeding multiple interconnected distal branches and draining veins. The aneurysmal form is most often caused by the erosion of an existing aneurysm into an adjacent vein.^[2,3]^ As the prevalence of rAVMs is <0.04%, rAVMs are a rare cause of hematuria.^[4,5]^ This can lead to clinical underdiagnosis and misdiagnosis of rAVMs. Therefore, after obtaining informed consent from the patient, we report a case of rAVMs and describe its diagnosis and treatment.

## 2. Case presentation

A 35-year-old female patient presented to a local hospital with right-sided lumbar abdominal pain with hematuria for 2 days. The pain was described as persistent colic. The patient also had nausea and vomiting with stomach contents and no other concomitant symptoms. A computed tomography (CT) scan of the abdomen suggested bleeding from the right renal pelvis and a catheter was placed to drain the bloody urine. The patient had a history of previous right ovarian cystectomy for 3 years, no history of renal injury, renal biopsy or percutaneous nephrolithotomy, and a history of allergy to aztreonam. There was no family history of hereditary disease. On admission, the patient had a temperature of 36.2 °C, a heart rate of 62 beats per minute, a respiratory rate of 17 breaths per minute and a blood pressure of 125/82 mm Hg. Physical examination showed percussion pain in the right renal area, no percussion pain in the left renal area, no signs of peritoneal irritation and no pressure pain in the ureteral travel area bilaterally. A catheter was in place, draining bloody urine. No other systemic abnormalities were noted. The patient was temporarily given symptomatic treatment such as hemostasis, anti-infection, and bladder irrigation.

**Figure 1.**
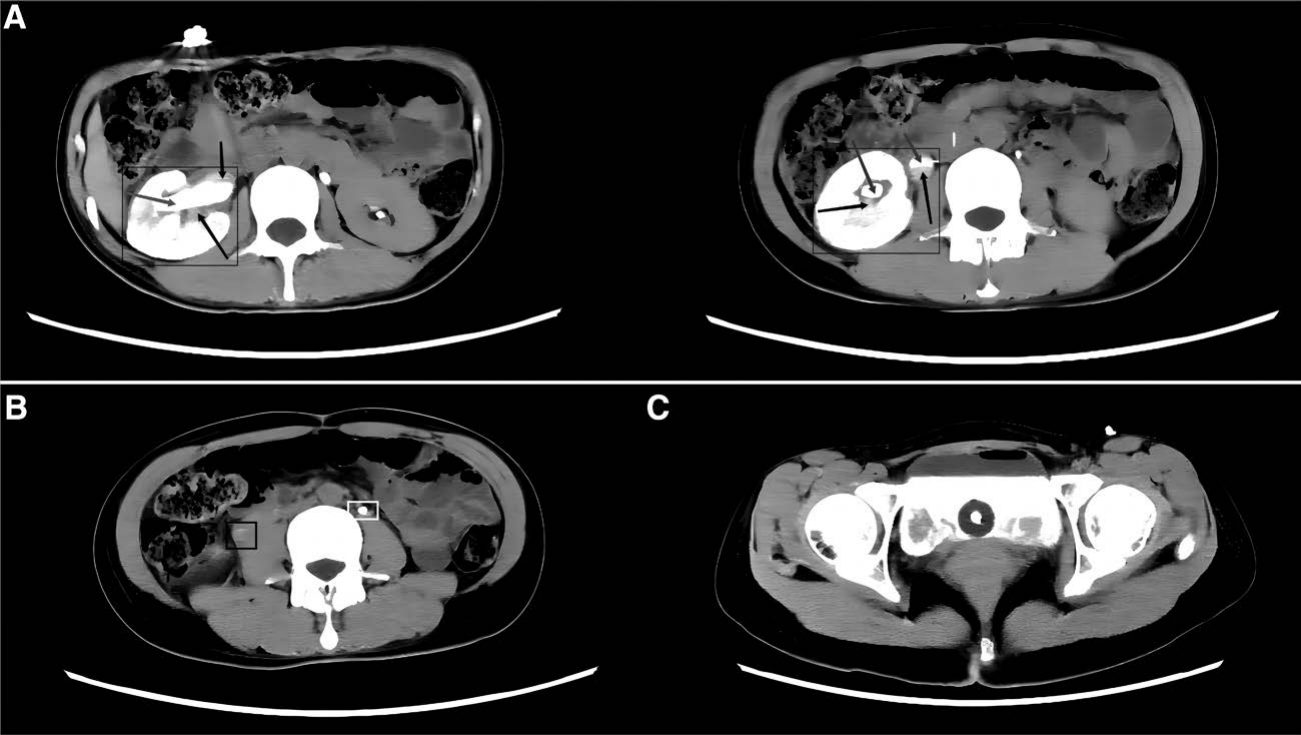
Computed tomography angiography. (A) Compared to the left side, the right renal pelvis and upper middle ureter are dilated and strips of white high-density shadow (gray arrow in black quadrant) and some gray low-density (black arrow in black quadrant) can be seen. (B) Compared to the left ureter (white quadrant), only a small amount of contrast filling is seen in this segment of the right ureter, and the lumen is mostly gray hypodense shadow. (black quadrant). (C) A large number of white high-density shadow and gray low-density shadow in the bladder can be seen.

Laboratory tests such as routine blood and blood biochemistry did not show any significant abnormalities. As shown on the urological CT scan. Streaks and patches of white high-density shadow (contrast) and some gray low-density shadow were seen in the right renal pelvis, in the middle and upper ureter and in the bladder lumen. Only a small amount of contrast filling was seen in the right ureteral segment, and the lumen was mostly gray hypodense shadow. This was considered to be an obstruction that resulted in dilatation of the renal pelvis and upper middle ureter (Fig. 1). Combined with the patient’s history, the gray hypodense shadow was an accumulation of blood, and this patient was considered to have an accumulation of blood in the right renal pelvis, upper middle ureter and bladder. The patient was bleeding heavily, so blood routine and coagulation routine were repeated. The results showed 2.16 × 10^12^ erythrocytes and 66 g/L hemoglobin. coagulation routine was not abnormal. Because of the low red blood cells and hemoglobin, 4 U of B-type de-white suspended red blood cells and 400 mL of virus-inactivated frozen plasma were transfused to the patient. The blood routine was reviewed, and the result showed 1.63 × 10^12^ erythrocytes and 49 g/L hemoglobin.

**Figure 2.**
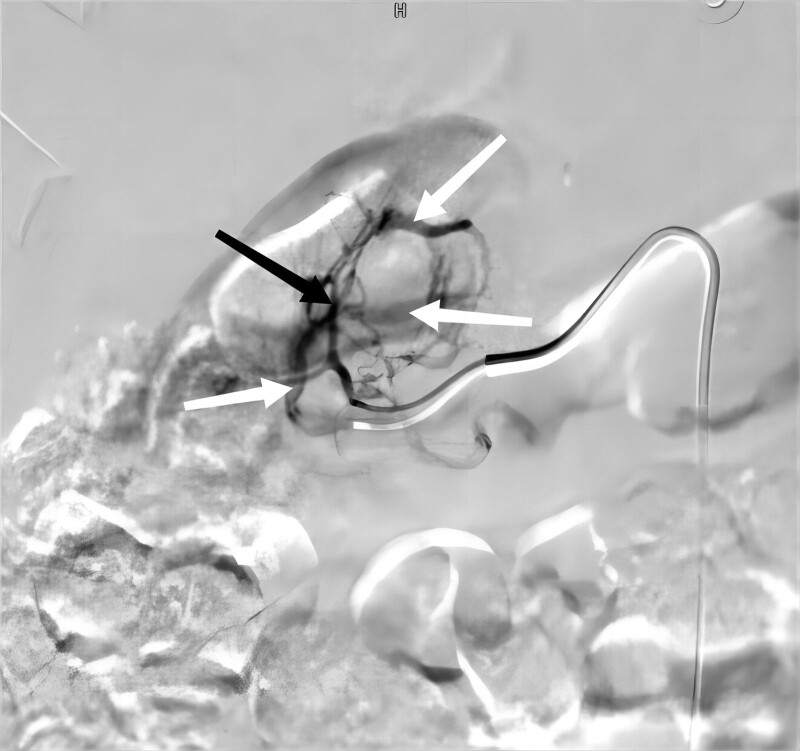
Renal angiography. A branch of the right upper pole supply artery is a tortuous supply artery (black arrow), which is connected to multiple thickened veins (white arrows).

**Figure 3.**
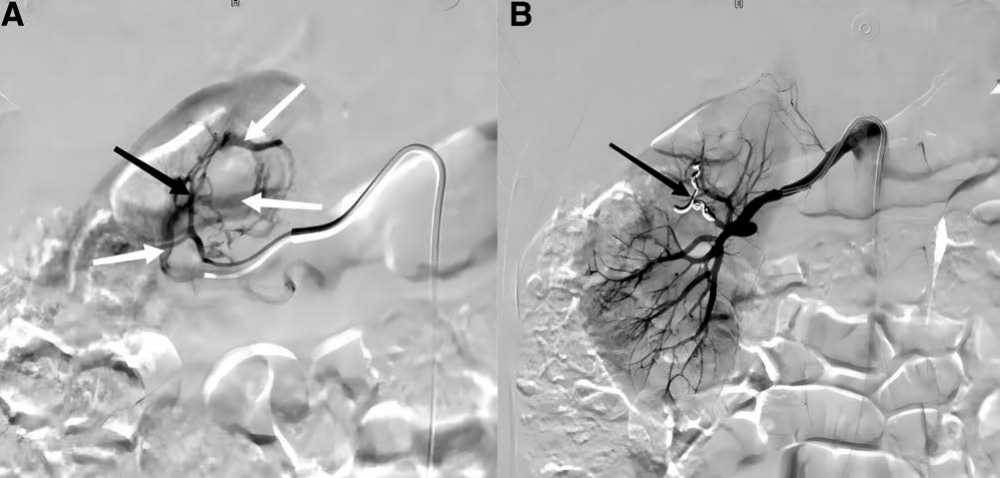
Renal angiography before and after management. (A) A tortuous supply artery (black arrow) and several thickened veins (white arrow) are visible; (B) the tortuous artery is obstructed and the thickened veins disappear after embolization.

The patient’s red blood cells and hemoglobin continued to decrease, and the patient was considered to be bleeding more and bleeding nonstop. In order to determine the cause of the bleeding and to stop the bleeding, it was decided to perform renal arteriography and renal artery embolization (RAE) of the renal artery at the same time as the transfusion according to the emergency operation. After ensuring that the patient met the surgical indications, the right femoral artery was punctured using the Seldinger method under local anesthesia and an arterial sheath was placed. A 5F pigtail catheter was placed in the abdominal aorta and angiography was performed. The angiogram showed a tortuous right upper renal pole branch artery and multiple thickened veins communicating with it and early visualization of the renal veins (Fig. 2). The intraoperative diagnosis was cirsoid rAVM. A Simon catheter was used with a microcatheter to insert the target vessel and 6 coils were slowly injected for embolization. On review of the angiogram, the tortuous right upper renal pole branch artery was found to be obstructed, and the thickened vein disappeared, and the renal vein was normally visualized in due course (Fig. 3). After the operation, 4 U of B-type de-white suspended red blood cells and 400 mL of virus-inactivated frozen plasma were transfused to the patient. The blood routine was repeated and the results showed 3.14 × 10^12^ erythrocytes and 94 g/L hemoglobin. The red blood cells and hemoglobin rebounded, and the patient’s condition was continued to be closely monitored thereafter.

On the third postoperative day, the patient’s urine was clear and there was no hematuria. Physical examination showed no percussion pain in the renal area, no pressure pain in the ureteral travel area, and no peritoneal irritation. These results indicated that the patient’s symptoms were significantly relieved. The patient was discharged from the hospital and followed up for 1 year. The patient gave feedback that she no longer had symptoms such as back pain and hematuria in her daily life.

## 3. Discussion

We report the case of a 35-year-old female patient with renal arteriovenous malformation whose main symptoms were right-sided lumbar and abdominal pain, hematuria and nausea, and vomiting. Digital subtraction angiography revealed tortuous branching vessels in the upper pole of the right kidney and prematurely visualized renal veins.

Hematuria, the main symptom of rAVMs, is caused by the rupture of diseased blood vessels lacking elastic fibers under the mucosa of the renal calyces or pelvis when vascular pressure is raised, causing blood to enter the collecting system.^[6]^ Blood entering the collecting system forms blood clots that can block the renal pelvis and ureter and cause pain in the lower back and abdomen.^[7]^ Therefore, rAVMs should be differentiated from diseases such as renal cell carcinoma and ureteral stones and other vascular abnormalities that cause hematuria and back pain. In addition to these symptoms, studies have also reported that rAVMs can cause hypertension and congestive heart failure.^[8]^

In clinical practice, ultrasound or CT is usually the first option for patients with hematuria and low back pain to rule out conditions such as renal tumors and ureteral calculi. In addition, cystoscopy is also often used to determine the source of the hematuria. When arteriovenous malformations (AVMs) are suspected, targeted angiography such as renal arteriography, abdominal CT angiography (CTA) or magnetic resonance angiography (MRA) should be performed. Renal arteriography is the gold standard for the diagnosis of rAVMs.^[6]^ This test directly reveals the location, number, blood supply, and hemodynamic changes of AVMs, including very small lesions, and allows treatment to be administered alongside the diagnosis. CTA and MRA have powerful 3D post-processing capabilities that allow clear visualization of the morphology, alignment and distribution of the renal vasculature from all angles. CTA and MRA are now also used to diagnose malformed vessels. Associated with the aneurysmal type. On CTA and MRA, rAVMs often appear as abnormally tortuous vessels located around the renal sinus, typically with enlarged blood supplying renal arteries and draining renal veins.^[9]^

Interventional treatment has the advantages of being less invasive, more effective and less recurrent with maximum preservation of normal kidney tissue.^[10]^ For symptomatic rAVMs, RAE is the best option.^[11]^ Surgery is less commonly used because it usually involves removal of the kidney, which carries the risk of loss of kidney function.^[10]^ In this case, we used an interventional approach to treat this patient.

For RAE, the presence of multiple fine blood vessels from different segments of the proximal portion of the renal artery is critical to the success of the treatment, so an assessment of the vascular structure is essential.^[1]^ In addition, an accurate assessment of the vascular structure is also useful in treatment planning and in the selection of embolization materials. coils, gelatin sponges, anhydrous ethanol, polyvinyl alcohol (PVA)and n-butyl 2-cyanoacrylate (NBCA) are often used as embolization materials in interventions. For embolization of cirsoid rAVMs, gelatin sponge, PVA and coil can be used alone, and liquid embolic materials such as NBCA and anhydrous ethanol can be used to achieve complete obstruction. For the treatment of cirsoid rAVMs, gelatin sponge, PVA, and coil embolization alone are prone to recurrence, however liquid embolic materials such as NBCA and anhydrous ethanol can achieve complete occlusion and have been widely used to treat this type of rAVMs. Liquid embolic material can also be used for angiomatous rAVMs. coils are mostly used for angiomatous rAVMs and aneurysmal rAVMs, again with the aid of NBCA to completely occlude the malformed vessel.^[12]^ When using liquid embolic agents such as NBCA, it is important to minimize parenchymal damage from embolization and to prevent the flow of embolic material into the systemic transvenous system. A balloon is placed in the renal artery and renal vein, respectively, to control flow and a balloon is placed in the artery supplying the malformed vessel for embolization. This prevents proximal embolic damage and prevents the material from entering the systemic veins.^[13]^Coils were used to embolize the tortuous artery and thickened vein in the tortuous right upper renal pole branch artery with good results. The patient was discharged from the hospital in good condition.

## 4. Conclusion

In summary, patients with rAVMs tend to present with hematuria and back pain. It is a rare disease and its presentation is similar to that of urinary tract tumors and stones, so an accurate diagnosis is essential. The preferred tests are ultrasound or CT, and the diagnosis is usually confirmed by CTA and digital subtraction angiography. rAVMs is often treated by endovascular embolization. The choice of material for embolization depends on the type of vascular malformation and the combination of materials used can improve the success rate of embolization. Care must be taken to avoid transfer of the embolization material during embolization.

## Acknowledgments

The authors would like to thank all those who contributed to this report and the patients who signed the informed consent form.

## Author contributions

**Data curation:** Xin Wang.

**Formal analysis:** Xin Wang, Zhankui Zhao.

**Writing – original draft:** Xin Wang.

**Writing – review & editing:** Zhankui Zhao.
